# The selective Cox-2 inhibitor Celecoxib suppresses angiogenesis and growth of secondary bone tumors: An intravital microscopy study in mice

**DOI:** 10.1186/1471-2407-6-9

**Published:** 2006-01-12

**Authors:** Frank Michael Klenke, Martha-Maria Gebhard, Volker Ewerbeck, Amir Abdollahi, Peter E Huber, Axel Sckell

**Affiliations:** 1 Department of Orthopedic Surgery, Inselspital, University of Bern, CH-3010 Bern, Switzerland; 2 Department of Experimental Surgery, University of Heidelberg, INF 365, D-69120 Heidelberg, Germany; 3 Department of Orthopaedic Surgery, University of Heidelberg, Schlierbacher Landstrasse 200a, D-69118 Heidelberg, Germany; 4 German Cancer Research Center, INF 280, D-69120 Heidelberg, Germany; 5 Department of Trauma and Reconstructive Surgery, Charité – Campus Benjamin Franklin, Hindenburgdamm 30, D-12200 Berlin, Germany

## Abstract

**Background:**

The inhibition of angiogenesis is a promising strategy for the treatment of malignant primary and secondary tumors in addition to established therapies such as surgery, chemotherapy, and radiation. There is strong experimental evidence in primary tumors that Cyclooxygenase-2 (Cox-2) inhibition is a potent mechanism to reduce angiogenesis. For bone metastases which occur in up to 85% of the most frequent malignant primary tumors, the effects of Cox-2 inhibition on angiogenesis and tumor growth remain still unclear. Therefore, the aim of this study was to investigate the effects of Celecoxib, a selective Cox-2 inhibitor, on angiogenesis, microcirculation and growth of secondary bone tumors.

**Methods:**

In 10 male severe combined immunodeficient (SCID) mice, pieces of A549 lung carcinomas were implanted into a newly developed cranial window preparation where the calvaria serves as the site for orthotopic implantation of the tumors. From day 8 after tumor implantation, five animals (Celecoxib) were treated daily with Celecoxib (30 mg/kg body weight, s.c.), and five animals (Control) with the equivalent amount of the CMC-based vehicle. Angiogenesis, microcirculation, and growth of A549 tumors were analyzed by means of intravital microscopy. Apoptosis was quantified using the TUNEL assay.

**Results:**

Treatment with Celecoxib reduced both microvessel density and tumor growth. TUNEL reaction showed an increase in apoptotic cell death of tumor cells after treatment with Celecoxib as compared to Controls.

**Conclusion:**

Celecoxib is a potent inhibitor of tumor growth of secondary bone tumors *in vivo *which can be explained by its anti-angiogenic and pro-apoptotic effects. The results indicate that a combination of established therapy regimes with Cox-2 inhibition represents a possible application for the treatment of bone metastases.

## Background

Angiogenesis – the formation of new blood vessels from an already established microvasculature – is a key contributor to the pathogenesis of several diseases such as benign and malignant tumors, rheumatoid arthritis, and diabetic retinopathy. A solid tumor cannot grow beyond a critical size of 1–2 mm^3 ^or metastasize without an adequate blood supply [1,2]. The development of a new vascular network consists of several steps which are controlled by various endogenous stimulators and inhibitors [3]. The best investigated endothelial cell specific growth factors are the vascular endothelial growth factor (VEGF) family and its receptors VEGFR-1, VEGFR-2, and VEGFR-3 [4,5]. Besides hypoxia which is the main inducer of VEGF, prostaglandins mediated by Cox-2 have been reported to regulate VEGF expression [6-8] which demonstrates an important link between Cox-2 activity and VEGF expression [9]. Cox-2 is induced by cytokines, growth factors and tumor promoters and is over expressed in inflamed and malignant tissues [10]. The enzyme is localized in neoplastic cells, endothelial cells, and stromal tissues [9,11-13] and contributes to tumor angiogenesis and tumor growth by (1) an increased expression of the angiogenic growth factor VEGF [14], (2) the production of eicosanoid products which can directly stimulate endothelial cell growth factor induced angiogenesis [15], and (3) the inhibition of tumor and endothelial cell apoptosis by up regulating the antiapoptotic protein bcl-2 [16,17]. Due to the multiple links between tumor angiogenesis, tumor growth and Cox-2 expression, selective pharmacological inhibition of Cox-2 represents a promising therapeutic strategy for the treatment of malignant solid tumors.

Celecoxib, a selective Cox-2 inhibitor has been shown to effectively decrease tumor angiogenesis and reduce tumor growth of a variety of experimental primary tumors including colorectal, prostate, and breast tumors [18-24]. However, the effects of Cox-2 inhibition on bone metastases which are found in up to 40% of autopsies of patients with a primary lung tumor [25] have not been described so far.

Here we report our *in vivo *findings that Celecoxib has anti tumor effects on secondary bone tumors of a non-small cell lung carcinoma by inhibiting angiogenesis and promoting tumor cell apoptosis.

## Methods

### Animal model and cell lines

Experiments were performed in 10 male adult severe combined immunodeficient mice (SCID, C.B-17/IcrCrl-scid-BR, Charles River Laboratories Inc., Sulzfeld, Germany, 7 to 8 weeks old, 20 to 25 g body weight), following institutional guidelines, approved by the local animal review board.

The human lung carcinoma cell line A 549 was obtained from the German Cancer Research Institute (Heidelberg, Germany). Tumor cells [1 × 10^7^/ml] were injected subcutaneously into the left flank of a donor mouse each and grown to a volume of 0.5 to 1.0 cm^3^. After sacrificing the donor mouse, the tumor was excised, cut into small pieces (volume 0.5–1.0 mm^3^) in Dulbecos Modified Eagle's Medium (DMEM,) at 4°C and implanted into the recipient mouse as follows.

All surgical procedures were performed in strictly aseptic conditions within a laminar flow unit (Merck Eurolab, Bruchsal, Germany) under deep anesthesia by an intra peritoneal injection of a mixture of ketamine (Ketanest^®^, 65 mg/kg body weight, Pfizer, Karlsruhe, Germany), xylazine (Rompun^®^, 13 mg/kg body weight, Bayer, Leverkusen, Germany) and acepromazine (Sedastress^®^, 2 mg/kg body weight, Medistar, Holzwickede, Germany). The surgical preparation was performed as described previously [26]. In brief, the scalp of the mouse was shaved and was surgically excised in an oval area to expose the frontal and parietal bone. The periosteum was removed and an oval cavity of approximately 2 by 1 by 0.5 mm was milled into the calvaria by eliminating parts of the external tabula of the calvaria including the spongious bone underneath. Then one piece (approx. 0.5–1.0 mm^3^) of the human lung carcinoma A 549 was implanted into the cavity. To prevent the tumors from dehydrating or from mechanical damage, the preparation was sealed with a glass cover slip and bone cement.

The animals were housed individually in special filter cages to maintain aseptic conditions and to prevent mutual damaging of the cranial window. The animals were provided with sterile standard pellet food and water *ad libitum*.

### Cox-2 inhibitor treatment

The selective Cox-2 inhibitor Celecoxib was a generous gift of Pharmacia Inc. (St. Louis, MO, USA). Celecoxib was dissolved in a carboxymethylcellulose (CMC)-based vehicle at 5 mg Celecoxib/ml vehicle. Five animals each were treated once daily by s.c. injection of 30 mg/kg body weight Celecoxib (Celecoxib, n = 5) or the equivalent amount of the (CMC)-based vehicle alone (Control, n = 5). Treatment started on day 8 after tumor implantation and was continued until termination of experiments on day 28 after tumor implantation.

### Intravital microscopy

For intravital microscopy, mice were anesthetized and positioned on a custom made stereotactic device.

Within the first week after tumor implantation, mice were observed daily under epi-illumination with a stereotactic microscope (Leica MZ7_5_, Leica, Germany) employing a 5 to 40 fold magnification. At 24 hour intervals, the first appearance of (i) hemorrhage, (ii) the first appearance of newly formed blood vessels entering the implanted tumor tissue, and (iii) the onset of perfusion in these newly formed vessels were determined. Two-dimensional tumor growth was determined *off line *by measuring the tumors surface area on days 7, 14, 21, and 28 after implantation.

Intravital fluorescence video microscopy was performed using an epi-illumination fluorescence microscope unit (Leica, Germany) equipped with a 4× (EF 4/0.12, Leitz, Wetzlar, Germany) and 40× (Zeiss Achroplan 40×/0.75 w, Carl Zeiss, Germany) objective on days 7, 14, 21 and 28 after tumor implantation. For *off line *analysis, regions of interest were recorded on video tapes using a S-VHS videocassette recorder (AG-7350, Panasonic, Japan) at a rate of 50 frames/s and a digital camera (Kappa CF 8/1, Kappa Opto-electronics, Germany).

Using an adequate fluorescence filter set for green light (bandpass 515–560 nm), the intravenous injection of fluorescein isothiocyanate (FITC)-labeled dextran (Sigma, St. Louis, MO, FITC-Dextran, FD 2000S, molecular weight 2.000.000; 0.1 ml of a 5% solution in 0.9% NaCl as a plasma marker) enabled the observation of the tumor microcirculation.

### Off-Line analysis of tumor growth and microhemodynamics

Tumor growth was determined *off-line *by measuring its two-dimensional surface area in mm^2 ^from standardized digital photographs of the cranial window preparation at 10–fold magnification on days 7, 14, 21, and 28 after implantation using a computer based analysis program (AnalySIS^® ^V3.0, Soft Imaging System, Münster, Germany).

The functional microvessel density (*FVD*) was determined as the length of all perfused microvessels within a tumor in relation to the two-dimensional surface area of the tumor in mm/mm^2 ^indicated by the fluorescence of FITC labeled dextran in all perfused vessels. Recordings on video tape for *off line *analysis of the functional vessel density were made for 15 s each. The *off line *analysis was performed using a computer based image analysis program (CapImage^®^, Engineering Office Dr. Zeintl, Heidelberg, Germany).

### Histopathologic assessment

At the end of the experiment, tumors were immediately excised with the surrounding tissue of the calvaria and the brain for further histopathologic investigation. Tissue samples were fixed for 24–48 hours by immersion in 4% formalin solution. After decalcification of the bone in ethylene-diaminetetraacetic acid for 2 weeks, samples were embedded in paraffin and sliced in three-μm serial sections for Hematoxylin-Eosin staining and five-μm serial sections for immunohistochemistry.

For the TUNEL reaction, the *in situ *Cell Death Detection Kit (Cat. No 1684809, Roche Diagnostics, Mannheim, Germany) was used according to the manufacturer's instructions: Briefly, deparaffinized sections were pretreated with proteinase K in 10 mM Tris/HCl and washed in phosphate buffered saline (PBS). Sections were then incubated with Triton 0.1% (Triton^® ^x-100, Lot. No. 93424, Fluka, Buchs, Switzerland), washed in PBS, and incubated with 5% BSA (Albumin from bovine serum, A-9647, Sigma, St. Louis, MO) to block endogenous peroxidase. For TUNEL reaction, the sections were incubated with the TUNEL reaction mix. Sections were then rinsed with tris buffered saline (TBS) and incubated in converter alkaline phosphatase, followed by TBS washings. Sections were then stained with Fast Red (Sigma FastTM, F-4648, St. Louis, MO) for 10–15 minutes depending on staining intensity. Sections were counterstained with Mayer's hematoxylin (Hämalaun Mayer, Art.-Nr. 1A-528, Chroma, Münster, Germany) and mounted with AquaTex (AquaTex^®^, Cat.No. 1.08562, Merck, Darmstadt, Germany). Treatment procedures for Control specimen were the same except for omitting the primary antibody.

For quantitative analysis of apoptotic cells, standardized digital images were acquired with a color digital microscopic camera system (Leitz Diavert microscope, Leica, Bensheim; AxioCam^®^, Carl Zeiss, Göttingen, Germany) with a resolution of 1300 × 1030 pixel at 200 fold magnification in three randomly distinguished regions of interest (ROI) of each slide and processed with AxioVision Rel. 3.1 software package (Carl Zeiss, Göttingen, Germany). The total number of tumor cells and the number of apoptotic tumor cells were counted. The percentage of positive cells with TUNEL staining served as rate of apoptosis.

### Statistics

All numerical data are presented as median with 25% and 75% quartile. Using the software program SigmaStat^® ^for Windows (Version 2.03, SPSS, Chicago, IL), data were analyzed statistically with ANOVA on ranks. Mann-Whitney Rank Sum Test was applied for pair wise comparison procedures. Differences were considered significant at p < 0.05.

## Results

The first newly formed vessels were observed 6 days after implantation in all tumors. Vessel formation was followed by a rapid onset of perfusion within the next 24 hours. Intravital microscopy showed that the origin of the angiogenic sprouting was from the vessels located within the surrounding bone.

**Figure 1:**
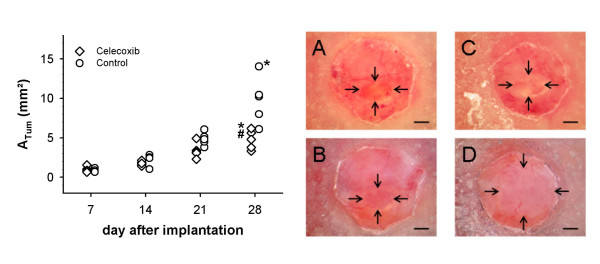
**Graph: **Tumor surface, time course. Y-axis: tumor surface (A_TUM _in mm^2^), x-axis: time in days after implantation. ANOVA on ranks (p = 0,016); *p < 0.05 versus day 7, ^#^p < 0.05 versus Control, Mann-Whitney Rank Sum Test. Significant increase in two-dimensional tumor surface in both groups between days 7 and 28 after tumor implantation. Two-dimensional tumor surface is significantly reduced in animals treated with Celecoxib compared to Controls on day 28. **A, B, C, D**: Photographs of the cranial window with A 549 lung carcinoma, top-view, scale bar 1000 μm. Tumor borders marked by arrows. Day 7: Celecoxib (A), Control (C). Day 28: Celecoxib (B), Control (D).

Within the first week after tumor implantation, tumor growth was identical in all animals. After the initiation of treatment on day 8 after tumor implantation, animals treated with Celecoxib showed a reduced tumor growth compared to Controls without significant differences between both groups until day 21. On day 28 after tumor implantation, the two-dimensional tumor surface was found statistically smaller with 4.8 mm^2 ^(3.7/5.7) in Celecoxib compared to 10.3 mm^2 ^(7.5/11.4) in Controls (Fig. 1).

**Figure 2:**
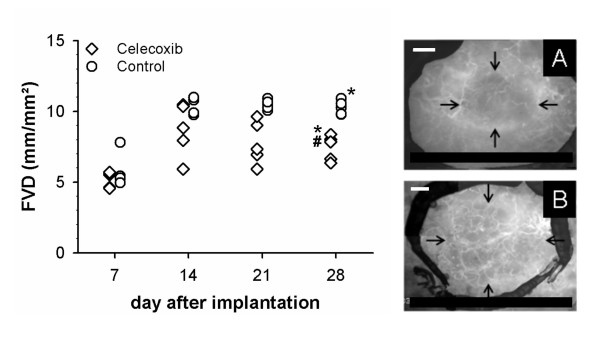
**Graph: **Functional vessel density, time course. Y-axis: functional vessel density (FVD in mm/mm^2^), x-axis: time in days after implantation. ANOVA on ranks (p = 0.008); *p < 0.05 versus day 7, ^#^p < 0.05 versus Control, Mann-Whitney Rank Sum Test. Significant increase in functional vessel density in both groups between days 7 and 28 after tumor implantation. Functional vessel density is significantly reduced in animals treated with Celecoxib compared to Controls on day 28. **A, B**: Photographs from intravital fluorescence microscopy, top-view, scale bar 200 μm. Tumor borders marked by arrows. Day 28: Celecoxib (A), Control (B).

Functional vessel density (FVD) in the Control animals increased between day 7 and day 14 after tumor implantation, reaching a constant plateau between days 14 and 28. Between days 7 and 14 FVD was similar in animals treated with Celecoxib. However, from day 21 after tumor implantation, FVD decreased in animals treated with Celecoxib. On day 28, FVD was significantly lower in Celecoxib treated animals compared to Controls (10.3 mm/mm^2 ^(9.8/10.6) vs. 7.8 mm/mm^2 ^(6.6/8.1), p = 0.008, Fig. 2). In both groups, FVD was significantly higher at day 28 after tumor implantation compared to the first measurements on day 7 after tumor implantation.

**Figure 3:**
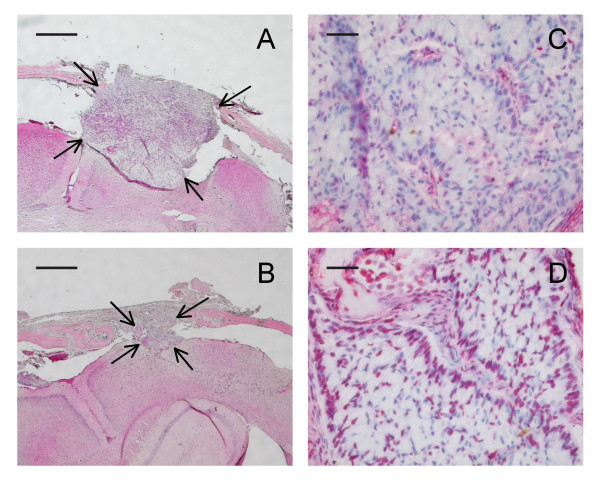
**A, B**: A 549 lung carcinoma at day 28, hematoxylin-eosin stained cross section, scale bar 500 μm. Tumor borders marked by arrows. Control (A), Celecoxib (B). **C, D**: A 549 lung carcinoma at day 28, TUNEL reaction, scale bar 50 μm, apoptotic cells stained red. Control (C), Celecoxib (D).

The growth of the implanted tumors was observed histologically in all of the cranial window preparations (Fig. 3 a,b). The tumor dimensions determined from the H&E stained tissue slices showed a close correlation to the tumor sizes quantified from intravital microscopy investigations. TUNEL staining showed an increased rate of apoptosis in tumors treated with Celecoxib (p < 0.001). The percentage of apoptotic cells per total tumor cell number was 54.4% (44.0/65.8) after Celecoxib treatment versus 21.8% (4.4/43.5) in Controls. Cell counting also revealed a reduced overall density of tumor cells with 3394 cells/mm^2 ^(3129/3936) in animals treated with Celecoxib compared to 4996 cells/mm^2 ^(4466/5732) in Controls (p < 0,001). (Fig. 3 c,d)

The injection of Celecoxib or CMC-vehicle alone was well tolerated, no difference in animal behavior or loss of weight was observed.

## Discussion

Today's therapy of bone metastases with surgical procedures, chemotherapy, and radiation is limited to palliative treatment in many cases. Since angiogenesis is essential for tumor growth, selective targeting of tumor vasculature is a promising strategy that might help to overcome limited therapy options in bone metastases. Although bone metastases occur in up to 40% of lung cancer patients [25], research on antiangiogenic therapy has mostly been focused on primary tumors. Compounds inhibiting Cox-2 are of special interest for antiangiogenic therapy because prostaglandins mediated by Cox-2 have been shown to be important contributors to VEGF dependent tumor angiogenesis [27]. Cox-2 inhibitors have already shown promising antitumor effects in several solid tumors [18,19,22-24], however, the effects of Cox-2 inhibition on bone metastases have not been described so far. The main goal of the current study was to determine the effects of the selective Cox-2 inhibitor Celecoxib on tumor growth and angiogenesis of secondary bone tumors of a human non small cell lung carcinoma *in vivo*.

Previous studies have suggested that the antitumor activity of Celecoxib can be attributed, at least in part, to the inhibition of tumor angiogenesis [9,16,28,29]. Liu et al. [16] showed that the selective Cox-2 inhibitor NS 398 did not directly effect tumor cell proliferation of PC-3 prostate carcinomas but suppressed tumor growth through a down regulation of VEGF mediated tumor angiogenesis.

In the present study, Celecoxib significantly reduced functional vessel density in tumors and tumor size by 25% and 53%, respectively, compared to Controls. It has been shown previously that the antiangiogenic potential of selective Cox-2 inhibitors is mainly mediated by Cox-2 dependent mechanisms. Cox-2 regulates angiogenesis primarily through the eicosanoid products prostaglandin E_2 _(PGE_2_), Thromboxane A_2 _(TXA_2_) and Prostacyclin (PGI_2_) [6,15,30-36]. PGE_2 _stimulates the expression of VEGF, the key regulator of angiogenesis [6,30,31]. Downstream angiogenic actions of PGE_2 _also include the induction of the matrix metalloprotinases MMP-2 and MMP-9 and the integrin α_v_β_3 _which is selectively expressed on endothelial cells [32-35]. TXA_2 _and PGI_2 _mediate angiogenic actions such as endothelial cell migration and VEGF up regulation [15,36]. Antiangiogenesis, induced by endothelial cell apoptosis may be mediated by Cox-2 dependent and Cox-2 independent mechanisms [17,21,37-39].

The mechanisms and the causal relation between Cox-2 inhibition, angiogenesis and tumor growth were not investigated within the scope of the present study. However, our data demonstrate an effective anti tumor action of Celecoxib. The results of the functional vessel density time course indicate that one mechanism underlying the anti tumor effect of Celecoxib is the antiangiogenic potential resulting in a reduction of feeding and draining tumor blood vessels. Interestingly, the time course showed that the functional vessel density decreased in animals treated with Celecoxib from day 14 after tumor implantation while the tumor surface increased overall. However, tumor growth was decelerated by Celecoxib, resulting in a relatively reduced tumor size of 53% compared to Controls, suggesting that the decrease of functional vessel density precedes a reduction of tumor size.

Liu et al. [16] further stated that tumor growth suppression by Cox-2 inhibition is achieved by a combination of direct induction of tumor cell apoptosis and decreased angiogenesis because NS 398 also enhanced tumor cell apoptosis, detected with the TUNEL reaction. Since then, the proapoptotic effect of Cox-2 inhibitors on tumor cells and endothelial cells, being mediated via various pathways has been proved in several *in vitro *and *in vivo *studies [17,21,37,38,40-46]. Cox-2 dependent mechanisms are based on a decreased production of PGE_2 _which is associated with the modulation of pro- and antiapoptotic factors such as Bcl-2 and the prostate apoptosis-response gene (PAR-4) [17,38]. Cox-2 independent mechanisms of apoptosis induction by Celecoxib include (*i*) bcl-2 independent blockade of Akt signaling by inhibition of 3-phosphoinositide-dependent protein kinase-1 (PDK-1) and (*ii*) a caspase-9 and apoptosis protease-activating factor-1 (Apaf-1) dependent but bcl-2 independent mitochondrial pathway [21,37,41-45]. The significance of the Cox-2 independent apoptosis pathways was substantiated by the creation of Celecoxib derivates lacking Cox-2 inhibitory activity being able to effectively induce apoptosis via the same mechanisms as the parental Celecoxib, such as the blockade of Akt activation [42-44]. In the present study, apoptosis of tumor cells was investigated using the TUNEL assay. Consistent with previous findings, a significantly higher percentage of apoptotic tumor cells were found in tumors treated with Celecoxib compared to Controls. The exact mechanism by which apoptosis is augmented is not established in this study. However, it may be suggested that it is similar to that reported previously, which may include Cox-2 dependent as well as Cox-2 independent pathways [17,21,37,38,40-45].

## Conclusion

The data presented demonstrate that Celecoxib is a potent inhibitor of tumor growth of secondary bone tumors *in vivo*. This can be explained by its antiangiogenic and proapoptotic effects. We believe that a combination of established treatment procedures of bone metastases such as surgery, radiation, and chemotherapy with cyclooxygenase 2 inhibition is promising to improve the success of therapy.

## Competing interests

The author(s) declare that they have no competing interests.

## Authors' contributions

FMK participated in designing the study, carried out the animal experiments, the histology and the TUNEL assay, interpreted the data, and drafted the manuscript. MMG and VE participated in designing the study and revising critically the manuscript. AA and PEH established the A549 lung carcinoma cell cultures and tumor cell suspensions and participated in revising critically the manuscript. AS conceived, coordinated, and designed the study, performed the statistical analysis, interpreted the data, and drafted the manuscript. All of the authors have read and approved the final manuscript.

## Pre-publication history

The pre-publication history for this paper can be accessed here:
